# Reinforcing the bulwark: unravelling the efficient applications of plant phenolics and tannins against environmental stresses

**DOI:** 10.1016/j.heliyon.2022.e09094

**Published:** 2022-03-12

**Authors:** Zahra Dehghanian, Khashayar Habibi, Maryam Dehghanian, Sajad Aliyar, Behnam Asgari Lajayer, Tess Astatkie, Tatiana Minkina, Chetan Keswani

**Affiliations:** a Department of Biotechnology, Faculty of Agriculture, Azarbaijan Shahid Madani University, Tabriz, Iran; b Department of Biotechnology, College of Agriculture, Isfahan University of Technology, Isfahan, Islamic Republic of Iran; c Department of Biotechnology, Faculty of Agriculture, University of Mohaghegh Ardabili, Ardabil, Iran; d Department of Soil Science, Faculty of Agriculture, University of Tabriz, Tabriz, Iran; e Faculty of Agriculture, Dalhousie University, Truro, NS B2N 5E3, Canada; f Academy of Biology and Biotechnology, Southern Federal University, Rostov-on-Don, 344090, Russia

**Keywords:** Phenolics, Tannins, Plant adaptation, Secondary metabolites, Environmental stresses

## Abstract

Phenolic compounds are plant secondary metabolites that play a vital role in plant resistance. They are mainly synthetized from the amino acid L-phenylalanine, which is converted to trans-cinnamic acid in a series of biochemical reactions. These compounds take part in the regulation of seed germination and cooperate in regulating the growth of plants, also taking part in defense responses during infection, UV exposure, injuries, and heavy metal stress. The aim of this review is to discuss the role of phenolic compounds in the interactions of plants with various stress factors, both biotic and abiotic with special attention to their antioxidant properties. Therefore, understanding the biochemical potential of the phenylpropanoid derivatives would be beneficial in sustaining the metabolic processes used by plants to thrive and endure under adverse conditions.

## 1 Introduction

The majority of environmental stresses influence the active oxygen species synthesis in plants, resulting in oxidative stress (Kodikara et al., 2020). Furthermore, there is mounting evidence that proves plants are subjected to environmental stress. The counterbalance between the generation of activated oxygen species and antioxidant quenching activity is disrupted, which often results in oxidative damage (Zhang et al., 2017). Many crop casualties are caused by environmental stress. There are various stresses, many of which are crop-or location-specific. Water, high salinity, extreme temperatures, mineral nutrient shortage, metal toxicity, herbicides, fungicides, temperature, incremented UV-B radiation, insect pests, fungi, and weeds are among them. Most environmental stresses seem to have at least some of their impact by inducing oxidative harm (Benhiba et al., 2015). As a result, the antioxidant defense mechanism of plants has attracted the attention of many researchers (Bhattacharjee, 2019). Studies predicted that such stresses impact the development of plants and lead to a reduction of plant products and yield by 70% and 50%, respectively (Samota et al., 2017). Therefore, it is critical to minimize yield casualties by increasing efficiency by a variety of methods, such as the use of plant bio-stimulant products and the incitement of plants' secondary metabolism (Shahzad et al., 2018a).

Plants must respond to changing environments due to environmental stresses, and the phenolics aggregation in plant cells, which are thought to be plants’ reconciled reaction to the damaging environmental circumstances (Lattanzio, 2021). Plant phenolics (polyphenols) are the most abundant type of metabolites (secondary) in plants, with significant morphological and physiological importance. These components are aromatic combinations with one or more hydroxyl groups that originate from the pathway of phenylpropanoid/shikimate and the malonate/polyketide acetate process, containing phenols (polymeric/monomeric) and the polyphenols (Yang et al*.*, 2018a). In addition to protecting from environmental stresses, the phyto-phenolics are essential for plant growth (Lattanzio, 2021), like defense from diseases and predators (Pacheco-Ordaz et al., 2018), generating the fruits and vegetables sensorial and color-related qualities (Owolade et al., 2017), and some vital compounds such as antioxidant, antimicrobial, and antiallergenic function (Castrica et al., 2019). Phenolic combinations (besides tocopherols) are possible antioxidants in plant tissues: lignin precursor, flavonoids, and tannins could act as reactive oxygen species (ROS)-preventing combinations (Sharma and Melkania, 2017).

## 2 Classification and structure

Phenolic combinations have one aromatic ring with one or more hydroxyl replacements bound to it and vary in structure from basic phenolic molecules to fully polymerized combinations, exhibiting considerable structural variety and are frequently referred to as polyphenols (Bravo, 1998).

Most phenolic compounds naturally appear as conjugates with mono- and polysaccharides, coupled with one or more phenolic groups, and may also exist as functional derivatives like esters and methyl esters (Naczk and Shahidi, 2003). Since phenols are a wide and diverse group of chemical combinations, they could be categorized in different ways according to the number of carbons in the molecule (Table 1 and Table 2) (Wagay et al., 2020).

**Table 1:** Chemical classification of plant derived secondary metabolites (Modified from: Tuladhar et al., 2021).

Number of carbon atoms	Structural	Group
6		Basic phenol and benzoquinones
7		Phenolic acids
8		Tyrosine derivatives; phenylacetic acids; acetophenones
9		Isocoumarins; chromones; coumarins; phenylpropenes; hydroxycinnamic acids
10		Naphthoquinones
13		Xanthones
14		Anthraquinones; stilbenes
15		Flavonoids; isoflavonoids
18		Neolignans; lignans
30		Biflavonoids
n		Lignins; catechol; melanins; flavolans

**Table 2:** Summarizing the functional role of various polyphenols and tannins in the management of different abiotic stresses in plants.

Phenolic acids	Function/location	References
Caffeic acid	Concentration-dependent root growth inhibition	(Alberto et al., 2012)
	Existent in the nodule wall-bound part	(Chakraborty and Mandal, 2008)
Cinnamic acid	Rhizobia-induced resistance to *Rhizoctonia* in rice	(Karakaş et al., 2017)
Ferulic acid	Influence rhizobial development	(Seneviratne and Jayasinghearachchi, 2003)
	Concentration dependent root growth inhibition	(Alberto et al., 2012)
	Rhizobia-induced resistance to *Rhizoctonia* in rice	(Karakaş et al., 2017)
Gallic acid	Rhizobia-induced resistance to *Rhizoctonia* in rice	(Karakaş et al., 2017)
*p-*coumaric acid	In Rhizobia, it stimulates IAA generation	(Mandal et al., 2009)
	Existent in root and nodule	(Chakraborty and Mandal, 2008)
	Influence rhizobial development	(Mandal et al., 2009)
	Concentration dependent root growth inhibition	(Alberto et al., 2012)
*p*-hydroxybenzoic acid	Chem-oattractants, have an effect on the host range of the interaction	(Fonseca-García et al*.*, 2021)
	It is found in the soluble portion of young nodules	(Chakraborty and Mandal, 2008)
	Concentration dependent root growth inhibition	(Alberto et al., 2012)
Protocatechuic acid	In Rhizobia, it stimulates IAA generation	(Mandal et al., 2009)
	Existent in the nodule wall-bound part	(Chakraborty and Mandal, 2008)
	Rhizobial development can be affected	(Seneviratne and Jayasinghearachchi, 2003)
Salicylic acid	Aggregation in alfalfa roots	(Mandal et al., 2010)
	Indeterminate nodulation is inhibited by exogenous application	(Bastianelli et al., 2010)
	Soybean early nodulation is inhibited by exogenous SA	(Mandal et al., 2010)
	Auto-regulates nodulation at the stage of infection thread creation	(Hayat et al., 2012)
	Systemic resistance caused by Rhizobacteria mediated	(Mandal et al., 2010)
Vanillin	Peanut nod gene inducer	(Mandal et al., 2010)
Tannins	As antioxidants	(Barbehenn and Constabel, 2011)
	Prooxidant activity	(Summers and Felton, 1994)
	Toxins	(Berger et al., 2012)
	Protecting plants from herbivores	(Dahija et al., 2014)
	Protecting plants from herbivores	(Dahija et al., 2014)

## 3 Plants' phenylpropanoid pathway

**Figure 1:**
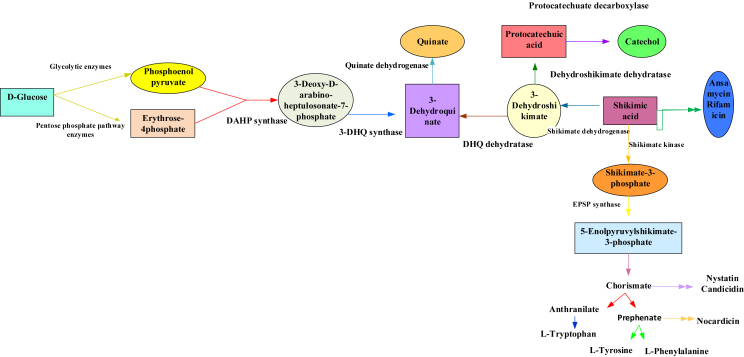
The shikimic acid or shikimate pathway (Modified from Ghosh et al., 2012).

**Figure 2:**
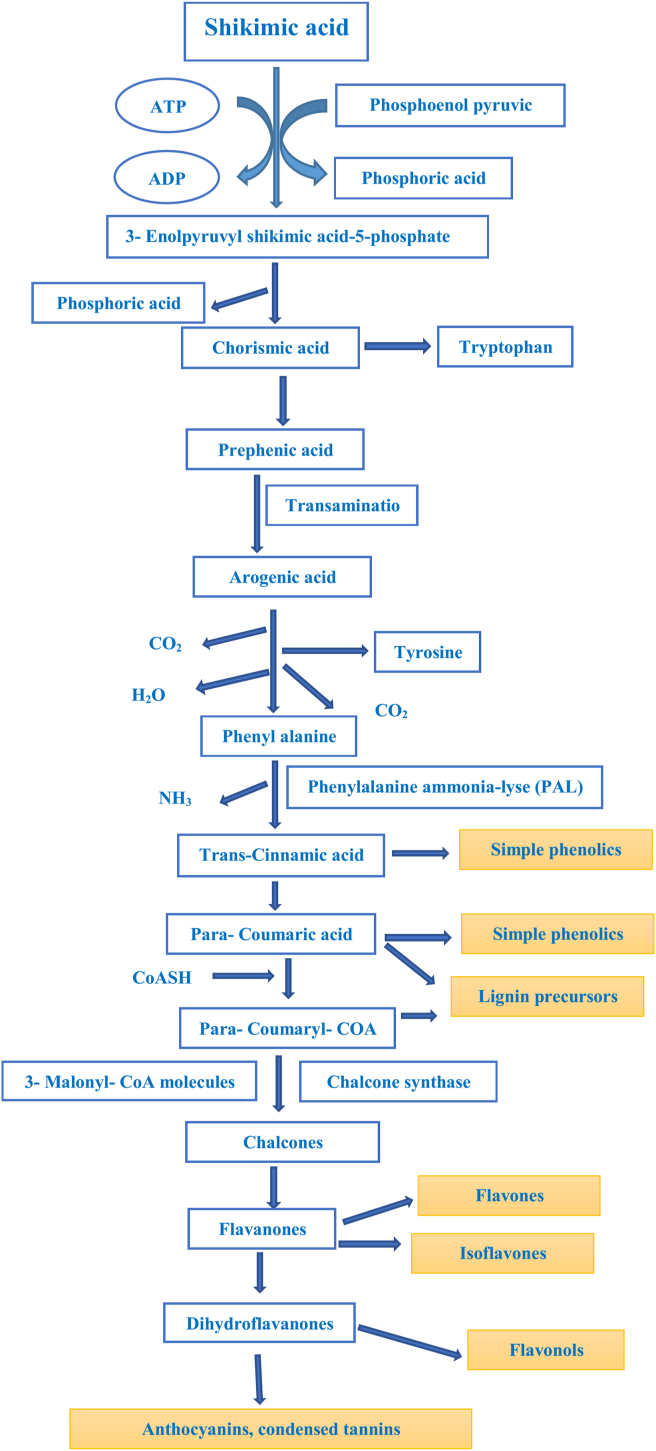
A schematic depiction of the phenolic combinations' biosynthesis in the phenylpropanoid and shikimate passages in plants (Naikoo et al., 2019).

Phenolics are a diverse group of molecules made up of around 10,000 combinations that are solvable in organic solvents and water (Yadav et al., 2018). According to Cheynier et al. (2013), phenolic biosynthesis via the malonic acid process is common in microorganisms such as bacteria and fungi but not in plants (Cheynier et al., 2013). However, the phenolic biosynthesis through the shikimic acid process is further widespread in bacteria, plants, or fungi and is not found in animals (Ayoub et al., 2016). The shikimic acid process utilizes precursors that originate from the cycle of pentose phosphate (Figure 1) (Bartwal et al., 2013) and glycolysis to generate various aromatic amino acids with phenylalanine as a repeated intermediary (Yadav et al., 2018). Phenolics are made up of a compound's variation, including stilbenes, styrylpyrones, arylpyrones, flavonoids, coumarins, tannins, lignans, and lignins (Khaleghnezhad et al., 2019). Phenylpropanoids are simple phenolic combinations made up of p-coumaric acid, cinnamic acid, and derivatives (Marienhagen and Bott, 2013). On the other hand, the phenylpropanoid groups in prominently branched polymers form a diverse group of phenolics (Figure 1 (Naikoo et al., 2019) and Figure 2 (Ghosh et al., 2012)).

Lignin is an example of complex phenolic compound that is extensively found in plants after cellulose (Yadav et al., 2018). Lignans are compositions made up of two molecules of a phenylpropene derivative present in the Asteraceae (for example, *Achillea lingulata* (Saeidnia et al*.*, 2011), Pinaceae (like *Cedrus deodara* (Pandita and Pandita, 2021), and *Rutaceae* (like *Fagara heitzii*) (Solyomváry et al*.*, 2017) families. Dibenzylbutane derivatives, dibenzyl butyrolactone (lignanolides or derivatives of butanolide), monoepoxy lignans or tetrahydrofuran derivatives, and bisepoxylignans or derivatives of 3,7-dioxabicyclo (3.3.0)-octane are the four principal subtypes. Most of these compounds had antibacterial and antifungal activity (Kandar, 2021), whereas others, including *wikstromal*, *matairesinol*, and *dibenzyl butyrolactol* from *Cedrus deodara* (Hussein and El-Anssary, 2019), showed cytotoxic activity.

Coumarins were made from the lactone of O-hydroxycinnamic acid, coumarin, benzo—pyrone. There has been a total of 1000 natural coumarins separated. Coumarin has been discovered in around 150 species from more than 30 distinct compounds. The most common source of coumarin is sweet clover (*Melilotus* spp.), Tonka bean (*Dipteryx odorata*), and Sweet Woodruff (*Galium odoratum*) (Oates, 2013). Coumarins like aesculetin, umbelliferone, and scopoletin are extant in plants as free compounds and glycosides (Pandita and Pandita, 2021). Coumarins had anti-inflammatory, anticoagulant, anticancer, and anti-pharmacological Alzheimer's effects (Xu et al*.*, 2015).

Flavonoids would be the most frequent kind of phenol found in nature. Since then, over 2000 of these compositions have been identified, with over 500 of them being found in their free condition (Mir et al*.*, 2020). A chroman circle with an aromatic circle in positions 2, 3, or 4 makes up the structural skeleton of flavonoids. Flavonoids were separated into different groups based on how much the central ring has been oxidized (ring C). Anthocyanins, flavones, and flavonols are the most prevalent. Flavones and their related compounds are frequently yellow (Latin flavus, yellow). They're found all over the place in nature, although they are most common in higher plants and immature tissues (in cell sap) (Sharma et al*.*, 2019c).

Although the dispersed group of plant secondary metabolites are present frequently in the heartwood of a wide range of plants, stilbenes are tiny. They are notably abundant in the heartwood of Pinus (*Pinaceae*) and Eucalyptus trees. The most common stilbene in nature is resveratrol, a para-hydroxylated composition. Resveratrol is found in *Picea*, *Pinus*, *Fabaceae*, *Myrtaceae*, and Vitaceae and has estrogen-like properties (Hussein and El-Anssary, 2019).

Plants that produce allelopathic phenolics can inhibit the development of plant species (Yadav et al., 2018). Phenolic combinations have some redox features that can serve as antioxidants and thus detoxify oxygen which is singlet (Huang and Bie, 2010). Phenolics have also been shown to greatly lower the risk of cancer in humans (Yang et al., 2018b). Phenolics also play other essential functions in plants such as nutrient uptake, enzyme activity, photosynthesis, and protein synthesis (Fayez and Bazaid, 2014). Phenols serve as stress index because their generation is higher in plants subjected to different stresses and harmful chemicals (Achakzai et al., 2009).

Plants contain phenolics as anti-herbivore constituents because their strong scent and taste deter animals and insects from grazing on them (Ashraf et al*.*, 2018). The major phenolic combinations, which interfere with plant development and defensive reaction toward microorganisms and insects, are flavones, anthocyanins, isoflavones, and flavonols (Yadav et al., 2018). Petal colors are caused by phenolic combinations like anthocyanins, which absorb pollinators (Khatiwora et al., 2010).

## 4 Tannins

### 4.1 The chemical composition of tannins

**Figure 3:**
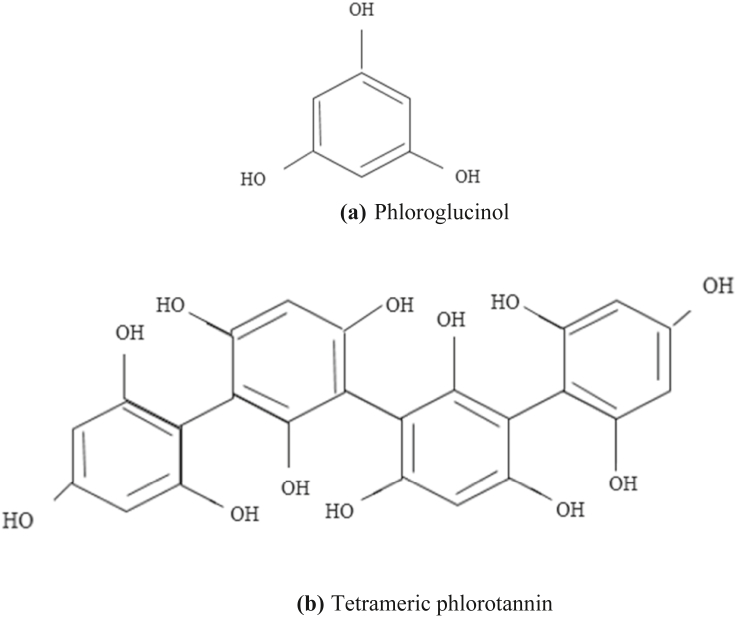
(a) phloroglucinol structures, the unit of the phlorotannin building, and (b) tetrameric phlorotannin consisting of four phloroglusinol units (Salminen and Karonen, 2011).

**Figure 4:**
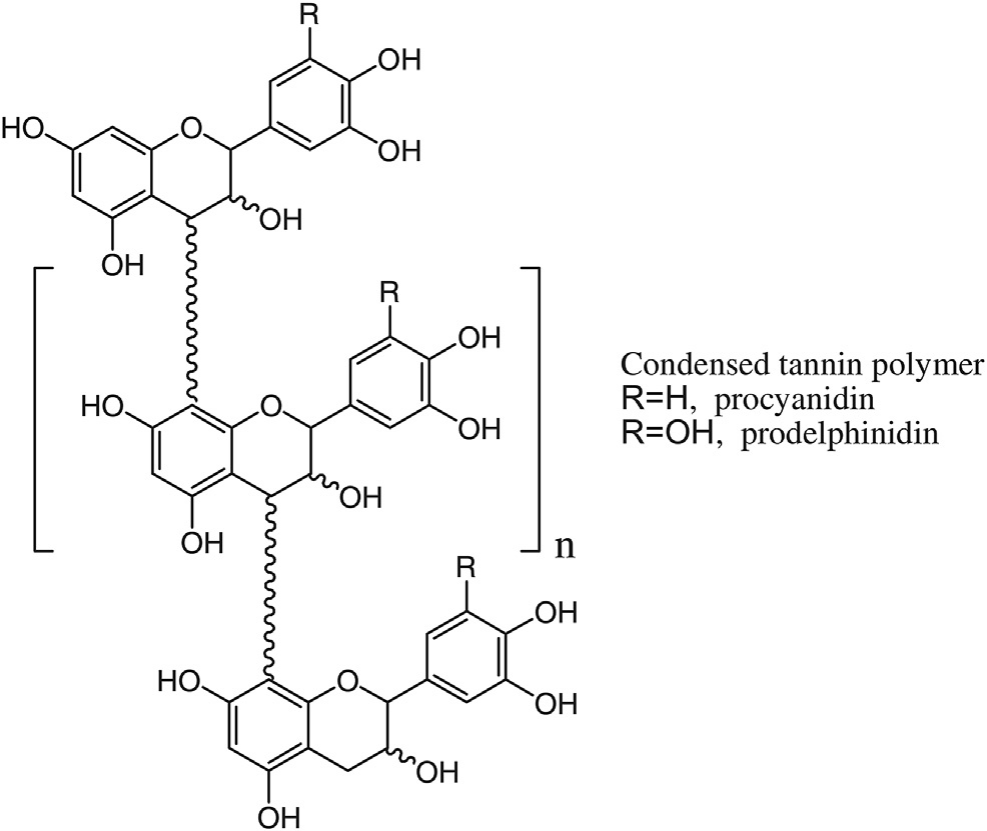
Condensed tannin polymer (Barbehenn and Constabel, 2011).

Tannins are the second most abundant polyphenol after lignins, and mainly they function as defense compounds that protect plants against pests and other abiotic stresses, such as drought, heat, and high UV radiation. Tannins can precipitate proteins and may reduce the activity of many enzymes. These compositions had been used to transform raw animal skins into leather for decades. Tannin molecules crosslink the protein, strengthening its resistance to bacterial and fungal attacks. Hence, recently, most compositions thought to be tannins have little, if any, ability to make leather due to their structure and biosynthetic origin (Girard et al., 2018). The three major classes of tannins are phlorotannins, condensed tannins (CTs), and hydrolysable tannins (HTs). Phlorotannins are mostly detected in aquatic species including brown algae (Mannino and Micheli, 2020); and structurally, they are the simplest tannin group. Single phlorotannins are made up of ≥2 phloroglucinol units (Figure 3a) linked together by C–O–C or C–C bonds, resulting in oligomers like tetrameric phlorotannin (Figure 3b). Increased OH-groups in the molecules or extra bonds among the monomers can result in structural changes. The most ordinary form of tannin is concentrated tannin, which is made up of two or more monomeric (-) epicatechin or (+)-catechin units (Figure 3a and b) (Clemensen, 2018). These types of CTs are named procyanidins (PC) (Figure 4).

**Figure 5:**
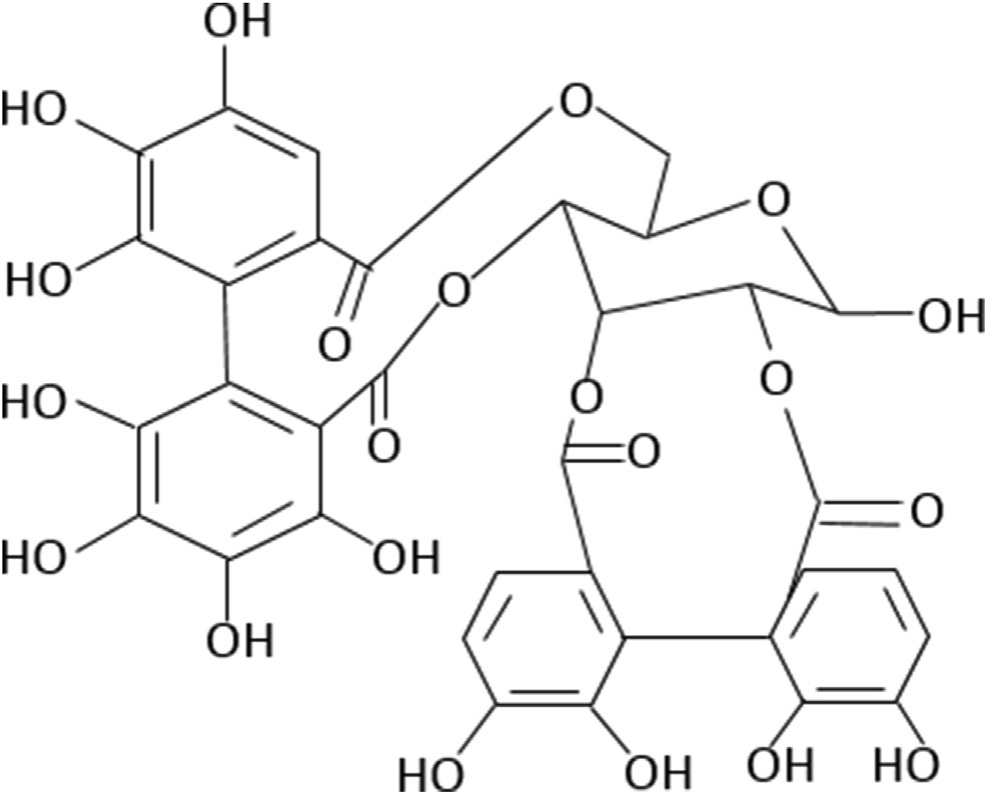
Chemical structure of pendunculagin (Hydrolysable tannins of walnut) (Amarowicz and Janiak, 2018).

The hydrolyzable tannins are secondary plant metabolites that are considered as phenolic combinations (Figure 5). Hydrolyzable tannins have been of interest in recent years because of their biological activity (Kumar Das et al., 2020). The hydrolyzable tannins are divided into three subclasses based on their structure: gallic acid compounds, gallotannins (GTs), and ellagitannins (ETs). GTs hydrolysis produces gallic acid, while ETs hydrolysis produces ellagic acid. Gallotannins are natural polymers that are generated by the succedent esterification of D-glucose and gallic acid hydroxyl groups in polymeric chains, with the galloyl moieties joined by so-called "depside" links. Polyphenols made up of two or more monoaromatic units joined by an ester bond are referred to as "depsides," and they contain a wide range of combinations (Barbehenn and Constabel, 2011).

**Figure 6:**
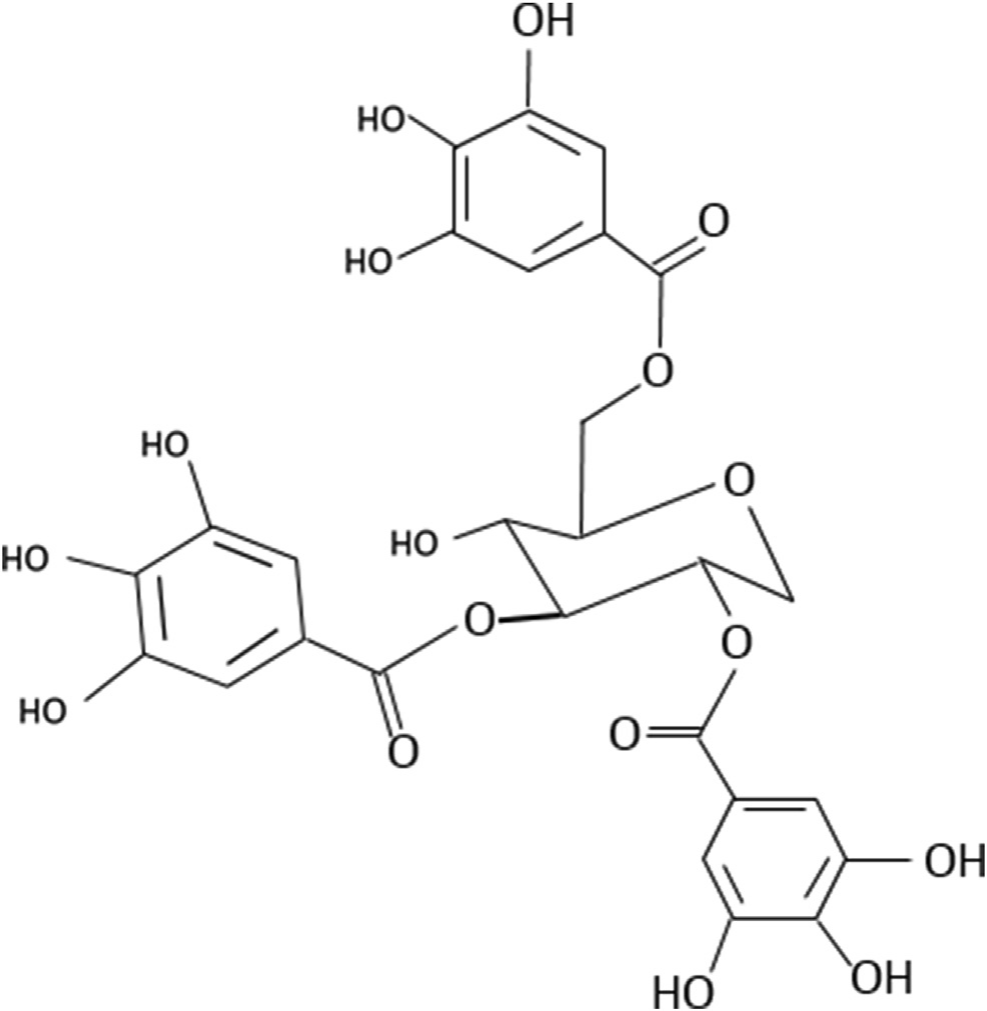
Chemical structure of glucitol-core containing gallotannins isolated from red maple (Amarowicz and Janiak, 2018).

**Figure 7:**
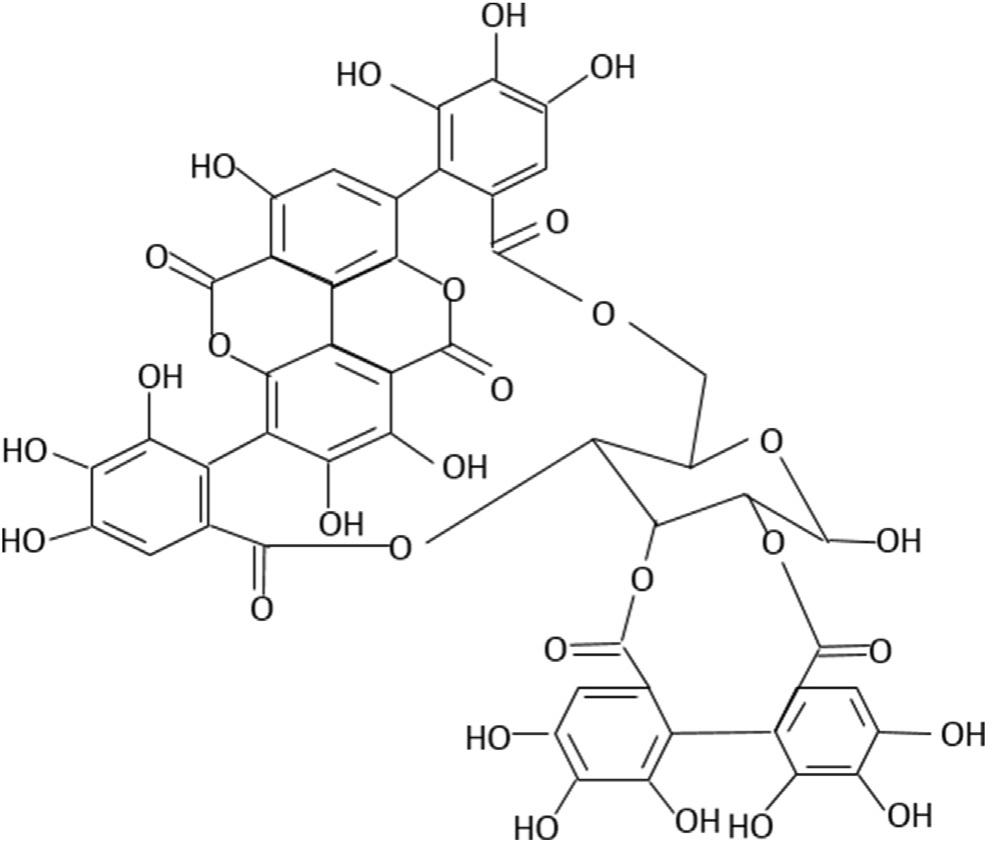
Chemical structure of punicalagin (Ellagitannins of pomegranate) (Amarowicz and Janiak, 2018).

The esterification of shikimic acid and quinic acid with gallic acid produces other forms of gallotannins. These gallotannins have one free carboxylic group in the quinic moiety and, like glucose-derived gallotannins, a variable number of depside bonds among their galloyl residues, resulting in a great structural variety (Karas et al., 2017). Galloyl groups are bound to a 1,5 anhydro-D-glucitol center in a glucitol-core containing gallotannins (GCGs) (Figure 6). Only maple *Acer* sp. such as the red maple, have yielded GCGs (*Acer rubrum*). Ellagitannins (ETs) are hexahydroxydiphenic acid esters and polyols such as glucose or quinic acid. After hydrolysis, the freed ET molecule from hexahydroxydiphenic acid is automatically reformed into ellagic acid, a water-insoluble combination. Methylation, glycosylation, and methoxylation all result in the formation of many ET compounds in plants (Figure 7) (Anstett et al*.*, 2019).

### 4.2 What is tannin?

Tannins are high molecular weight phenolic compositions, varying from 500 Da to 3000 Da present in plants’ leaves, barque, fruit, wood, roots, and mostly in the vacuoles. Tannins are linked to plant defensive processes against insects, birds, and mammalian herbivores (Hassanpour et al., 2011). These components are dissolved in water (a temperature range of 20–35 °C), except for certain higher molecular weight structures. Oligomeric compositions with free groups of phenol groups may form many complexes with minerals, starch, proteins, and cellulose. Tannins are present in the plants, which are flowering and non-flowering. Tannins are present in a lot of plants, including *Acacia* spp., *Sericea lespedeza*, and *Lotus* spp. (Hassanpour et al., 2011). While similar phenolic compositions including flavonoids, neolignans, and simple phenolics are defined and categorized based on their chemical structures, tannins are the diverse composition category that is largely linked by their capability to protein complexes (Hassanpour et al., 2011). Therefore, tannins are categorized as polyphenolic compounds that dissolve in water and can bind with the proteins to form soluble/insoluble tannin protein collection. Tannins can form complexes containing polysaccharides (hemicelluloses, pectin, and cellulose) as well as saponins, alkaloids, nucleic acids, and hormones (ChaichiSemsari et al., 2011). There are several findings showing the existence of tannins. For example, tannins are present in the vacuole of plant cells (Lagrange, 2020), and this has been proposed as a way of preventing tannins from inhibiting cell metabolism (Mir et al., 2015). Secondary metabolism, according to Mir et al. (2015), serves to sustain primary metabolism in situations unfavorable for development. Many recent studies have shown that tannins have affirmative impacts on animals by antimicrobial, protein bypassing, and anthelmintic impact on ruminants (ChaichiSemsari et al., 2011; Hassanpour et al., 2011).

### 4.3 Tannin biosynthesis in plants

Gottlieb (1990), who gave a straightforward and brief description of plant metabolism, reported that the main metabolism of autotrophic plants incorporates photosynthesis and respiration, from CO_2_ to the Lynen spiral's fatty acids (a reversible pathway) or the Krebs cycle's simple aliphatic acids using the Calvin cycle's sugars, pyruvic acid, and acetic acid, and then back to CO_2_. The acetate-malonate and shikimic acid passages are the basic polyphenolic synthesis metabolic passages in plants. For example, Hassanpour et al. (2011) discovered that one of the essential materials for the CTs synthesis is a flavan-3, 4-diol, and the second one is normally a flavan-3-of functioning like a nucleophile. CTs biosynthesis in the plants could be influenced by a variety of factors. Hassanpour et al. (2011) proposed that since polymers, which are isolated from the same plant root and leaf, exhibit structural differences, the biosynthesis of tannin may be regulated differently in the two specific tissues. Two distinct biosynthetic mechanisms of tannins in *L. pedunculatus* mutants were discovered: (1) light mediated, happening in the meristem (apical), and (2) nutritional, happening in the zone of the root.

The biosynthesis of CTs in the leaves is regulated by the quality of light, and in the zone of roots with stressing the plants by exerting nitrogen shortage situations. These contain plant species (Bharathidhasan, 2018), plant part (Hassanpour et al., 2011), plant maturation (Lagrange, 2020), developing season (Kumar et al*.*, 2020a, b), and the fertility of soil (Roca-Fernández et al*.*, 2020). Tannins are present in almost all herbs, shrubs, fruits, and legumes on the earth. There are important studies including on sorghum (Guzatti et al*.*, 2019), pomegranate (Santino et al., 2017), tea, wine (Hassanpour et al., 2011). Furthermore, recent studies discovered elevated CT levels in Acacia trees (e.g., *A. pycnantha, A. saligna, A. mearnsii, A. decurrent, A. dealbata*) (Waghorn, 2008).

### 4.4 Antimicrobial properties of tannins

Tannins have antimicrobial properties that can have a variety of biological and ecological consequences. Tannins have been shown to suppress the development of a wide range of microbes and filamentous fungus in pure culture, with the least inhibitory doses ranging from 0.5–20.0 g/l for bacteria and as low as 0.012 g/l for fungi (Njokuocha, 2020). Many kinds of microbes, such as human pathogens, free-living soil microbes, plant pathogens, food rotting saprophytes, and rumen symbionts, have been shown to have antimicrobial properties (Constabel et al., 2014). Microbes' susceptibility to tannins varies significantly depending on the microbial strain and the particular tannin (Tamkutė et al., 2021). Anderson et al. (2012) discovered that many CTs and HTs had different inhibitory activities on *Campylobacter jejuni* cells. CTs' effects may be reduced by adding casamino acids to the medium, suggesting that CTs operate on these bacteria by binding to proteins, but HTs act in a different way. According to this research, tannins suppress microbial growth, although the impact differs depending on the bacterial type (Jonker and Yu, 2017). Still, the kind of tannin matters when it comes to antimicrobial action. Purified *Desmodium ovalifolium* and *Myrtus communis* CTs, for example, are more inhibitory than the frequently used quebracho tannin. This in vitro research with pure bacterial cultures provides the most direct evidence of tannins' antibiotic activity. It must be noted that tannins' influence may appear otherwise in various populations, as observed in vivo, or ecologically related conditions. Yarahmadi et al. (2017) demonstrated that, while *Lotus corniculatus* CTs inhibit the development of numerous types of proteolytic rumen bacteria, total proteolytic activity remains constant. The processes through which tannins produce antimicrobial properties are unclear. Many studies show that tannins reduce the dissociation of cellulose, pectins, or hemicelluloses, which may involve the binding or deactivation of secreted enzymes or substrates. Higher-molecular-weight tannins induce proportionately more inhibitory in certain situations, showing that protein-binding is implicated. Tannins harm a wide range of enzyme activity in vitro (such as cellulases, pectinases, peroxidases, etc.) (Njokuocha 2020). In the absence of tannins, rumen bacteria's proteolytic function is decreased (Rivera-Méndez et al., 2017). Tannins could be used by certain microbial species in addition to their inhibition effect. Some *Staphylococcus*, *Klebsiella*, and *Bacillus species*, and certain fungal species (*Aspergillus niger*), can be capable of growing with tannin as their main carbon source. The generation of tannase, which acts on gallotannins, confers this capability (Ascacio-Valdés et al., 2014). Most tannins' antimicrobial effects in vitro implies that they may play a role in plant pathogen response. In a common garden study, Martínez-Arias et al. (2019) discovered a substantial connection between naturally variable tannin levels in *P. tremuloides* clones and tolerance to a Venturia shoot blight. The observed increase of the CT path in hybrid poplar following infection with *Melampsora medusae* supports a potential function for CTs in pathogen defense (Bai et al., 2020); and *Taphrina pruni*, a pathogen, excitation CT and chlorogenic acid production in plum (*Prunus domestica*) fruit (Gong et al., 2021). Takahashi et al. (2010) discovered a link between tolerance to the fruticolous fungus (*Ciboria batschiana*) and increased concentrations of tannins in *Quercus serrata* acorns.

Tannins have been extensively studied in a variety of plants, in which they are generally present in vegetative tissues such as roots, bark, and leaves. Tannins are frequently found in the bark of conifers, and the existence of CTs in the heartwood of *Acacia* sp. is well established (Constabel et al., 2014). Tannins are frequently observed in leaves, roots, and reproductive structures in plants. Tannin aggregation is often restricted to specific cell kinds. For example, vanillin found in subepidermal cells of both abaxial and adaxial surfaces of fresh *O. viciifolia* leaves (Constabel et al., 2014). The production of CTs moves from the abaxial to the adaxial side of the leaf throughout growth and into specialized cells termed idioblasts in the epidermis (Constabel et al., 2014). The bottom epidermis cells and the leaf mesophyll cells stain for CTs in new leaves. Tannin may be found in large quantities in the roots of certain woody plants, and root turnover adds to tannin buildup in the soil (Constabel et al., 2014). Tannins were found in a distinct area between the root tip and the suberized zone in *Pinus banksiana* and *Eucalyptus pilularis* (Bernjak and Kristl, 2020). Tannins seem to be accumulated in the walls of cortical cells after cell death. Since the specific roles of tannins in this root region are unknown, they merit further investigation. CTs were detected by DMACA labeling in the outer epidermal cells of the root tip of the camphor tree (*Cinnamomum camphora*), where they assist in resistance to Al toxicity. Tannin buildup caused by stress may indicate that tannins have extra functions in vegetative tissue, including leaves. This is especially true for *P. tremuloides*, where leaf CT levels improve after injury and insect herbivory (Rubert-Nason and Lindroth, 2021). According to molecular analysis, these increments are the consequence of fast transcriptional activation of the whole CT biosynthetic path in response to stress, and the PtMYB 134 transcription factor plays a major role in this activation (James et al., 2017). Increased tannin buildup is also caused by high visible-light exposure and nutrient deficiency (Najar et al., 2014). All these reactions might point to stress prevention role, possibly against reactive oxygen species (ROS), which are frequently produced during plant stress responses. The DkMYB 2 CT controller reacts to injury in *Diospyros kaki*, together with the CT biosynthetic genes, indicating that *D. kaki* CTs could be inducible (Yang et al., 2020).

Plant reproduction propagules, especially seeds and fruits, could be a great source of tannins (Sharma et al., 2019b). Since fruit tannins are essential in human diets, giving flavor and supporting health, there are numerous thorough experiments on the qualitative and quantitative features of tannins in fruit and seeds in food science research (Tzima et al., 2014). Tannin compositions and amounts have been widely studied in fruits, including grapes, apples, and blueberries (Grace et al., 2019). Tannins are abundant in the seed coats of *Phaseolus vulgaris* and other beans, especially colored types (Duan et al., 2021). Sorghum and barley seeds have CTs, while maize, rice, and wheat seeds do not (Shoeva et al., 2016). Premature seeds in high-tannin sorghum cultivars include flavan-3-ols, which polymerize pending seed maturity (Constabel et al., 2014). PAs are oxidized and polymerized into brown pigments in Arabidopsis seed coats that are sedimented on cell walls (Demonsais et al., 2020). CTs in the seed coat can aid in defending the embryo and endosperm from biotic stresses by acting as physical and/or chemical barriers, and via primary germination by preventing the passage of signaling molecules that alter seed dormancy (Zhou et al., 2021). High tannin concentration in cowpea seeds corresponds with resistance to bruchid beetles during storage (Kpoviessi et al., 2021). Rowanberry, blackcurrant, saskatoon berry, and sea buckthorn, as well as numerous Vaccinium species, have high CT concentrations (de Araujo et al., 2021). In a study of 99 food plants, approximately half of the species evaluated had tannins, primarily berries and fruit, but vegetables did not have observable tannins (MiljkoviĆ et al., 2018). Among the 33 food products examined by Nemes et al. (2018), considerable quantities of ellagitannins were found in 5 berry species. Tannin concentration in grape (*Vitis vinifera* L.) berries have been extensively studied, as tannins contribute to the flavor and stability of wines. The CTs and flavan-3-ol amounts are greatest in grape skins and grains; procyanidins predominate in grains, though prodelphinidins are plentiful in skins (Silva et al., 2020). It should be noted that tannin amounts may vary greatly across fruit types and cultivars (Dong et al., 2021). Certain fruits, such as the persimmon, have both high- and low-CT varieties. Low-tannin fruits stop generating CTs at a primary stage of growth, whereas tannin-rich fruits accumulate them until they are fully matured, imparting an unpleasant flavor (Zheng et al., 2021). Despite a wealth of research on the compounds and amounts of tannins in commercially important fruits, only a few species have been studied to see how they change as the fruit matures. It is commonly thought that the unpleasant taste of premature fruit is produced by greater tannin concentrations or changes in tannin solubility or composition during different growth stages.

## 5 Phenolics' physiological roles in plants

Phenolics are abundantly spread and play an important role in plant metabolism and physiological processes (Kumar et al., 2019). Phenolics affect different kinds of processes (physiological), which are related to plant development and extension, such as germination of seed, division of the cell, and the synthesis of the photosynthetic pigments (Sharma et al., 2019a). Specifically, a significant number of secondary metabolites with antioxidant activities (Sharma et al., 2019a) improve plant's efficiency under stress situations. Secondary metabolites enable plants to communicate with their surroundings. The microbes of soil convert phenolics into substances that facilitate nitrogen mineralization and humus formation (Halvorson et al., 2009). Moreover, phenolics increase nutrient absorption by chelating ions, which are metallic, increasing the active uptake sites, and increasing porosity of soil by rapid elements mobilization such as manganese (Mn), magnesium (Mg), iron (Fe), calcium (Ca), zinc (Zn), and potassium (K) (Perera and Tirimanne, 2021). Recently, an investigation discovered, Zn usage and treatment of the PGPRs (plant growth-promoting rhizobacteria) increased the organic acids (oxalic acid, pyruvic acid, methylmalonic acid, malic acid, oxaloacetic acids, succinic acid, malonic acid, tartaric acid, and citric acid), and phenolics content in wheat root exudates, which aided in Ca, N, and Zn nutritious mobilization and absorption (Rehman et al., 2018).

Phenolic combinations also help in the fixation of nitrogen in legumes (Blum, 2019). Plant phenolics prevent catabolism of the IAA (dihydroxy B-ring flavonoids) and restrict synthesis of the IAA (monohydroxy B-ring flavonoids) as a physiological controller or chemical messenger (Weston and Mathesius, 2013). Flavonoids are important in the production of useful pollen (Falcone Ferreyra et al*.*, 2012). For example, Tohge et al*.* (2018) reported that adding a little flavonol aglycone kaempferol or quercetin dose to matured pollen during pollination restores fertility. Due to cellular enzyme activity disturbance and cell division impairment, certain phenolic combinations (p-hydroxybenzoic acid, benzoic acid, coumarin and trans-cinnamic acid) might be potentially phytotoxic if stored in large amounts and could impede the development of seedlings and germination (Shuab et al., 2016). High phenolic acid content, on the other hand, has been linked to improved seed germination (Mandal et al., 2010). Polyphenol-rich spruce bark extractive increased germination while inhibiting root development in *Lycopersicon esculentum* (Sharma et al., 2019a). Phenolics decreased seed tegument thickness and improved seed tegument porosity, which aids water absorption and increases germination (Alam et al*.*, 2020). In maize and sunflower, polyphenolic extractive of spruce bark increased photosynthetic rate and biosynthesis of assimilative pigment (chlorophyll a/b) (Tanase et al., 2014). Phenolics altered the composition of thylakoids and mitochondrial membranes, lowering the energy needed for ion transfer (Suksungworn et al*.*, 2021). Phenolic combinations serve as antioxidants by inhibiting ROS, catalyzing oxygenation reactions by the metallic complexes production, and preventing oxidizing enzymes' functions (Weidner et al., 2009).

## 6 Impacts of environmental stresses

### 6.1 Abiotic stress resistance and phenolics

#### 6.1.1 Phenolic compounds as sunscreens (ultraviolet)

In open fields, plant's exposure to atmospheric radiation of UV-B (280–320 nm) harms DNA, proteins, and membranes, as well as alters metabolism with ROS generation. Plants generate phenolic compositions that function as a screen within the layer (epidermal) of the cell to protect the tissues against harmful radiation, as well as regulating the antioxidant mechanisms in the cellular and whole-organism levels. Thus, the process prevents mutations and cell destruction caused by units of the thymine dimerization in DNA and the coenzymes NAD or NADP potential photo-degradation (Daayf and Lattanzio, 2009). The flavonoids are effective UV screens due to their high absorbance at 250–270 and 335–360 nm (Lattanzio, 2021). The flavonoids and the phenolic compositions have an essential function in UV protection (Sembi et al*.*, 2019; Neugart et al., 2021).

A change in the amount of flavonoids compound in plant leaves is caused by light abundance or radiation of the UV, which primarily activates biosynthetic genes of the flavonoid (Doupis et al., 2016). Several experiments have shown that excessive sunlight and radiation of UV alters the leaves of plant flavonoid content (Doupis et al., 2016). Several scholars have identified polyphenols as plant protective processes to counteract UV radiation as direct shields and critical biological feature (Reboredo and Lidon, 2012). According to Larcher (1995), phenolics, specifically anthocyanin that aggregate in the epidermis, will serve as a filter that is darkened and defends the mesophyll from severe radiation. During stress, flavonoids (kaempferol derivatives particularly), isoflavonoids or phenolic acid esters, and psoralens aggregate UV-B from entering the mesophyll (Tamrat Alemu and Roro, 2020). Flavonols and flavones, the major types of flavonoids present in the flowers, aggregate in the leaves, stems epidermal layers, and grab light intensely in the zone of UV-B without interfering with wavelengths of observable PAR, to protect cells from severe radiation of UV-B (Lake et al., 2009). After exposure of the UV-B, the number of flavonols in *Picea abies* (Norway spruce) increased (Hammerbacher et al., 2020). UV light has been shown in many plant species to transcriptionally activate the expression of CHS, and it's a primary enzyme in the biosynthesis process of flavonoids (Kumar and Ellis, 2003).

#### 6.1.2 The role of phenolics in the stress of drought

Drought stress is the main significant nonliving stress that influences plant development and results in yield reduction. Plants' phenolic amount is enhanced when water is scarce. Flavonoid aggregation was reported by Nakabayashi et al. (2014) to be important to increase drought resistance in wild and mutants of *Arabidopsis thaliana*. The content of quercetin (a type of flavonol) increased substantially in white clover during drought situations, with increased levels in drought-tolerant genotypes (Ballizany et al., 2012). Under drought conditions, Alirezalu et al. (2018) reported that flavonol levels increase in many plants, including *Crataegus monogyna* and *Crataegus laevigata*. An increase in flavanol amounts was also observed in *Cistus clusii* plants under regulated treatments of drought, as well as in plants harvested in a crop field in summer, which was marked by heat-zone temperatures and a sustained rain deficit in the Mediterranean region (Hernandez et al., 2004). According to Akula and Ravishankar (2011), the defensive system to drought stress is activated by the leaf phenolic molecules bioactivity. Plants respond to drought stress by accumulating antioxidants and sun shields via phenolic acids and flavonoids (Nichols et al., 2015). Larson (1988) stated that elevated levels of phenolic acids and flavonoids in willow leaves cause oxidative stress in drought situations. According to Sánchez-Rodríguez et al. (2011) drought-tolerant tomato cultivars had higher levels of quercetin and kaempferol. The radical scavenging potential of black-hulled and red-hulled rice was determined by anthocyanins and proanthocyanidins amounts, respectively (Atawodi et al*.*, 2010). Under drought conditions, wheat leaves produce significant amounts of flavonoids and phenolic acids, as well as cell-damaging oxidants (Ma et al., 2014). According to Nichols et al. (2015), the quercetin, flavonols, and kaempferol elevated amounts were associated with increased stress tolerant ability of white clover plant toward drought stress.

#### 6.1.3 The function of phenolics in salt stress

Salt induced conditions cause the development of ROS such as hydrogen peroxide, ions of hydroxyl, and superoxide anions (Acosta-Motos et al., 2017), which necessitates a well-organized and plant antioxidant tuned mechanism activation to counteract ROS proliferation (Martinez et al., 2016). Phenolic compositions have strong antioxidant activity and help in the toxic ROS detoxification in plants that are exposed to salinity stress (Chen et al., 2019a). Furthermore, the phenylpropanoid biosynthetic process is activated in response to salty stress, resulting in the generation of various phenolic compositions with high antioxidative capacity (Bistgani et al., 2019). Some specific genes, including the VvbHLH1 gene, are implicated in the increased generation of flavonoids by controlling biosynthetic pathway genes and giving salinity tolerance to plants (Golkar and Taghizadeh, 2018). Under salt stress, NtCHS1 functions as a critical action in the flavonoids biosynthesis in tobacco plants, which accumulates straightly in ROS scavenging (Chen et al., 2019b). Flavone biosynthesis also increases under salt stress; and salt stress increases the flavone synthase gene expression, GmFNSII-2, and GmFNSII-1, in *Glycine max* (Yan et al., 2014). Anthocyanin biosynthesis was reported to be enhanced in plants grown in salt situations (Ben Dkhil and Denden, 2012).

#### 6.1.4 Function of phenolics in heavy metal stress

Metal stress induces oxidative stress in plants by causing the damaging ROSs development, which can lead to toxicity and development retardation (Shahzad et al., 2018b). Furthermore, in plants, increased phenolic biosynthesis under metal stress aids in the protection of plants against oxidative stress (Handa et al., 2019). Flavonoids may improve the chelation of metal mechanism, that aids to reduce the toxic hydroxyl radicals' amounts in plant cells (Almughraby et al., 2019), and this fact is well-matched with the finding that metal abundance increases the flavonoids amounts in plants (Handa et al., 2018). Metal toxicity promotes the aggregation of various flavonoids implicated in assisting the plant's defensive system, such as anthocyanins and flavonols (Kısa et al., 2016). Agglomeration of phenolic compositions is caused by the up regulation of phenylpropanoid enzyme biosynthesis, which includes cinnamyl alcohol dehydrogenase, shikimate dehydrogenase, phenylalanine ammonia-lyase, polyphenol oxidase, and chalcone synthase (Chen et al., 2019a), based on the transcript levels modulation of genes that encode biosynthetic enzymes (Leng et al., 2015). Flavonoids are noted for their H_2_O_2_ scavenging ability (Keilig and Ludwig-Mueller, 2009). The G6PDH (glucose-6-phosphate dehydrogenase) and SKDH (Shikimate dehydrogenase) are essential enzymes that accelerate the biological function needed for the generation of phenylpropanoid process precursors (Kováčik et al., 2009).

Cinnamyl alcohol dehydrogenase (CADH) is another enzyme that catalyzes biochemical processes that generate precursors for lignin synthesis (Mishra and Sangwan, 2019). In plants, heavy metals activate the biosynthetic process for phenylpropanoids by increasing the essential biosynthetic enzymes activity such as G6PDH, CADH, SKDH, and PAL (Mishra and Sangwan, 2019). Furthermore, polyphenol oxidase (PPO) helps in the ROS mechanism of scavenging and increases the plant's tolerance to nonliving stress factors including heavy metals (Kováčik et al., 2009).

### 6.2 Biotic stress

#### 6.2.1 Resistance to disease

Most plant phenols, or secondary metabolites are generated in larger quantities and function as phytoalexins, nematicides, and phytoanticipins aimed at soil pathogens and insects, which are phytophagous (Chitara et al., 2020; Kumar et al., 2021; Singh et al., 2021; Tripathi et al., 2021). Plants manufacture phytoalexins such as hydroxycinnamate conjugates and hydroxycoumarins to protect themselves from a variety of disease-causing pathogens.

When plant saps are attacked by microorganisms and get injured, "reaction zones" emerge (Kemp and Burden, 1986). This effect has been found in plants such as cucumber, soybean, peach, tobacco, and cotton (Kumar et al., 2020a, b). As plant pattern diagnosis receptors recognize possible pathogens based on their conserved pathogen-associated molecular pattern, phenolic compositions are synthesized, leading to PAMP-triggered immune. Thus, the progression of the disease is halted before the pathogen gains full control of the plant (Singh et al., 2014; Keswani 2015; Ram et al., 2019). In some cases, the plants developed immunity due to prior disease or wound. In a study on beech plants conducted by (Preminger, 2019), it was observed that the bark of beech trees that have been injured previously had more phenolic concentration than sensitive beech trees. Only a few other naturally tolerant plants have a higher phenolic content than the others. Compared to sensitive species like peach, almond, and apple, rot-resistant plant roots like persimmon, pecan, and passionfruit have more content of phenolic combinations. Phenolic combinations including protocatechuic acids, ferulic acid o-coumaric acid, and p-coumaric acid function in a similar way of preventing fungal development (Perradin et al., 1983). Other causes, including the enzymes needed in phenolic compositions biosynthesis, may be accountable for disease tolerance, like therapies that reduce or inhibit certain enzymes (Chalker-Scott and Fuchigami, 2018).

#### 6.2.2 Response to herbivores

Phenolic compositions protect plants against herbivores by invading ruminant gut flora (Cotas et al*.*, 2020). Herbivores can consume a large range of plants (Champagne et al., 2020). Tannins can precipitate proteins and can inhibit certain enzymes’ action. Tannins, as seen in insects, function by decreasing the protein amount in the diet; hence, tannins are recognized as decreasing digestibility combinations (Champagne et al., 2020). Condensed tannins (CTs) could prevent the enzymes (extra-cellular) formed by the bacteria. Tannins also impart a bitter flavor to the leaves of the plant, ultimately repelling herbivores (Kubalt, 2016). The digested hydrolyzable tannins (HTs) phenolic compounds attraction may have negative consequences on invertebrates, like harming kidney and liver (Champagne et al., 2020).

## 7 Conclusion and future prospects

The biosynthetic pathway for polyphenolic compounds, also known as the phenylpropanoid pathway, is the most studied and tested secondary metabolite generation pathway in the Plantae kingdom. Furthermore, their concentration increases when plants are under biotic or abiotic stress. This defensive effect of polyphenols is ascribed to the capability of ROS scavenging as well as defend the plants from extreme sunlight. These combinations are used for a variety of purposes, like antioxidants, allelochemicals and plant development enhancement (Bujor et al., 2015). Phenols and polyphenols influence the supply and flow of inorganic and organic soil nutrients available for plants and/or microbes. They also show a sensitive response to nutrient deficiency, thus providing a method for diagnosing nutrient disorders prior to the appearance of visible symptoms, also act as information carriers and play a vital part in the initiation and integration of the symbionts with the plants. Because of the plethora of advantages provided by phenolic compounds, they have been proposed to serve as alternatives to the chemicals used for control of pathogens in the agriculture sector. These compositions can be used in the formulation of cosmetics that act on pathologies or conditions harmful to the skin, which increase the interest in the use of these bioactive molecules in pharmaceutical products. Phenolic compounds are an alternative to artificial food additives and can be interesting for the development of functional foods that aim to maintain good health. However, there are still some gaps that need to be better studied so that the use of these compounds can reach a large industrial scale and for the administration and consumption of them to be effective and efficient to guarantee their benefits without causing a side effect. Still, for applications in the food industry, methods to increase the stability and to preserve the bioactivity of these compounds during the useful life of food products must be investigated.

## Ethical approval

This article does not contain any studies with human participants or animals performed by any of the authors.

## Code availability

Not applicable.

## Consent for publication

All authors have provided the consent to jointly publish this review article.

## Declarations

### Author contribution statement

All authors listed have significantly contributed to the development and the writing of this article.

### Funding statement

This work was supported by the Ministry of Science and Higher Education of the Russian Federation project on the development of the Young Scientist Laboratory (no. LabNOTs-21-01AB) and by the Strategic Academic Leadership Program of the Southern Federal University (“Priority 2030”) to Chetan Keswani.

### Data availability statement

No data was used for the research described in the article.

### Declaration of interests statement

The authors declare no conflict of interest.

### Additional information

No additional information is available for this paper.
